# DEPTOR loss impairs brown adipocyte development *in vitro* but has limited impacts in mice

**DOI:** 10.1016/j.molmet.2022.101660

**Published:** 2022-12-16

**Authors:** Charles Colas, Mathilde Mouchiroud, Manal Al Dow, Alona Kolnohuz, Yves Gélinas, Alexandre Caron, Mathieu Laplante

**Affiliations:** 1 Centre de recherche de l'Institut universitaire de cardiologie et de pneumologie de Québec - Université Laval (CRIUCPQ), 2725 Chemin Ste-Foy, Québec, QC, Canada, G1V 4G5; 2 Centre de recherche sur le cancer de l’Université Laval, Université Laval, 9 rue McMahon, Québec, QC, Canada, G1R 3S3; 3 Faculté de Pharmacie, Université Laval, 1050 avenue de la Médecine, Québec, QC, Canada, G1V0A6; 4 Département de Médecine, Université Laval, 1050 avenue de la Médecine, Québec, QC, Canada, G1V0A6

**Keywords:** DEPTOR, mTOR, brown adipocyte, brown adipose tissue, Adipogenesis, Thermogenesis, Cold exposure

## Abstract

**Objectives:**

The mechanistic target of rapamycin (mTOR) is a serine/threonine kinase that regulates growth and metabolism. In mice, activation of mTOR controls cold adaptation by promoting the recruitment and the activation of brown adipose tissue (BAT). DEP-domain containing mTOR-interacting protein (DEPTOR) interacts with mTOR to modulate its activity. Whether DEPTOR levels are modulated by cold in BAT and whether this protein regulates brown adipocyte development and thermogenic activation has never been tested.

**Methods:**

DEPTOR levels were measured in mouse tissues upon cold exposure and in brown preadipocytes following the induction of adipogenesis. Lentiviruses expressing short-hairpin RNA were used to repress DEPTOR expression in brown preadipocytes *in vitro*. Conditional deletion of DEPTOR in brown preadipocytes and in mature brown fat cells was achieved by crossing DEPTOR floxed mice with either *Myf5-*Cre or *Ucp1-*Cre^ERT2^ mice. These animals were exposed to cold and extensively phenotyped.

**Results:**

DEPTOR is highly expressed in BAT and its levels are induced by chronic cold exposure, a condition that triggers BAT expansion and activation. Supporting a role for DEPTOR in brown fat cell recruitment, we found that DEPTOR is induced during brown adipocyte development and that its depletion impairs adipogenesis *in vitro*. This adipogenic lesion was associated with defects in both Akt activation and the expression of key adipogenic regulators. Conditional deletion of DEPTOR in brown preadipocytes or mature brown fat cells did not impact BAT recruitment and thermogenesis in mice but slightly reduced the expression of adipogenic and lipogenic genes.

**Conclusions:**

DEPTOR is highly expressed in BAT and its levels are dynamically regulated during brown fat cell development and upon cold exposure. Although DEPTOR depletion severely represses brown fat adipogenesis *in vitro*, its deletion is dispensable for BAT development, recruitment, and thermogenic activation in mice.

## Highlights

• DEPTOR is highly expressed in brown adipose tissue.
• DEPTOR expression is dynamically regulated in response to cold.
• DEPTOR levels are induced upon brown adipocyte differentiation.
• DEPTOR depletion represses adipogenesis *in vitro*, but not *in vivo.*

## 1 Introduction

Brown adipose tissue (BAT) serves as a key heat-producing organ that impacts body temperature in mammals [1,2]. Upon cold stimulation, activation of the sympathetic nervous system (SNS) acutely activates triglyceride breakdown, fatty acid oxidation and thermogenesis in BAT. Chronic cold exposure also triggers a well-defined transcriptional program required to sustain heat production in brown adipocytes [3]. BAT thermogenesis depends on the uncoupling protein 1 (UCP1), a mitochondrial protein that deviates protons across the inner mitochondrial membrane, uncoupling oxidative phosphorylation from ATP synthesis [1,2]. As a result, energy is dissipated in the form of heat. For a long time, BAT was thought to be present only in small mammals and human newborns. However, positron emission tomography analyses revealed that BAT is also present and active in adult humans [4,5]. Several studies now suggest that BAT may be a valuable target to improve glucose metabolism and insulin sensitivity in humans [[6], [7], [8], [9], [10], [11], [12]].

The mechanistic target of rapamycin (mTOR) is a serine/threonine kinase that controls several biological processes to promote anabolism, growth, and proliferation [13]. This kinase is part of two complexes termed mTOR complex 1 (mTORC1) and 2 (mTORC2). In response to nutrients and growth factors, mTORC1 phosphorylates various effectors to increase the synthesis of the macromolecules needed to support growth. On the other hand, mTORC2 triggers the phosphorylation of AGC kinases, including protein kinase B/Akt (Akt), to promote metabolism and survival [13]. Over the last years, the mTOR signaling pathway was reported to impact BAT function in rodents. Studies from independent groups showed that mTORC1 activity is highly induced by acute and chronic cold exposure and β3-adrenergic receptor stimulation [[14], [15], [16], [17], [18]]. Interestingly, BAT denervation completely blocks cold-mediated mTORC1 activation, confirming that SNS stimulation is sufficient to activate mTORC1 *in vivo* [15]. Supporting the importance of mTORC1 in BAT development and function, loss of mTORC1 in adipocytes reduces BAT size and completely prevents cold-induced BAT expansion, mitochondrial biogenesis, and oxidative metabolism in mice [15,16,19]. The role of mTORC2 in BAT has also been extensively studied in mice. Reports show that mTORC2 is activated by acute cold but repressed by chronic exposure to low temperature [14,15,17,18,20]. Loss of function studies revealed that the mTORC2/Akt axis impacts BAT function by promoting glucose uptake, glycolysis, *de novo* lipogenesis, and adipogenesis [14,17,[20], [21], [22]]. Altogether, these findings position the mTOR pathway as a central node regulating BAT activation and thermogenesis in mice.

DEP-domain containing mTOR-interacting protein (DEPTOR) was identified in 2009 as a novel factor interacting with both mTORC1 and mTORC2 [23]. Because DEPTOR binds and represses mTOR kinase, this protein was originally characterized as an inhibitor of both complexes. However, manipulating DEPTOR expression in cells and tissues showed that the impact of DEPTOR on mTOR signaling is complex and non-linear [24]. Because many negative feedback loops emerge from mTORC1 to repress the activity of phosphoinositide 3-kinase (PI3K) [25,26], an upstream and dominant regulator of mTORC2, DEPTOR-mediated inhibition of mTORC1 often activates mTORC2 and its downstream effector Akt [24]. For this reason, DEPTOR is now considered as a modulator of mTOR signaling rather than a simple mTOR inhibitor [24].

The ability of DEPTOR to rewire the mTOR signaling pathway has been shown to affect several metabolic processes in mice including the regulation of energy balance [27], liver metabolism [28], and white fat cell development [29]. However, the impact of DEPTOR on BAT development and function has never been tested. Here, we identify BAT as one of the tissues showing the highest expression of DEPTOR in mice. We show that DEPTOR levels are dynamically regulated in BAT upon acute and chronic cold exposure. Studies *in vitro* indicate that DEPTOR is highly induced during brown adipocyte development and that its depletion impairs brown preadipocyte differentiation. The adipogenic defect linked to DEPTOR loss is associated with impaired Akt activation and lower expression of key adipogenic regulators. Conditional deletion of DEPTOR in brown preadipocytes or mature brown fat cells did not impact BAT recruitment and thermogenesis in mice but slightly reduced the expression of adipogenic and lipogenic genes. Our observations indicate that DEPTOR plays a positive role in brown fat adipogenesis *in vitro*, but that its expression is dispensable for BAT formation, recruitment, and thermogenic activation in mice.

## 2 Material and methods

### 2.1 Cell culture

T37i cells were isolated from a mouse hibernoma [30]. Immortalized brown preadipocytes were kindly provided by Dr. Shingo Kajimura [31]. Sub-confluent cells were maintained in Dulbecco's Modified Eagle Medium (DMEM) with 10% fetal bovine serum (FBS).

### 2.2 Virus production and infection

The hairpins used to repress DEPTOR expression in cell culture experiments were obtained from the RNAi Consortium (TRC). The identification numbers and the sequences of these hairpins are: sh_Ctrl [TRCN0000072246, CAAATCACAGAATCGTCGTAT], sh_Deptor_2 [TRCN0000110157, CGCAAGGAAGACATTCACGAT], sh_Deptor_4 [TRCN0000110159, GCAAGGAAGACATTCACGATT] and sh_Deptor_6 TRCN0000110158, GTCGGAAATCTACCAGCTTTA]. Briefly, pLKO.1 vectors containing the shRNA sequence were co-transfected in 293T cells with psPAX2 and pMD2G. Virus-containing supernatants were collected at 48 h after transfection and filtered using a 0.45 μm filter. Cells were infected for 24 h in the presence of 8 μg/ml polybrene. After infection, the cells were dispersed into fresh medium. Cells were selected on the following days with 1.5 μg/ml puromycin. The expression of *Deptor* mRNA and DEPTOR protein was measured by qPCR and western blot respectively to validate the knockdown efficiency.

### 2.3 Adipogenesis *in vitro*

Immortalized brown preadipocytes were induced to differentiate using an adipogenic cocktail containing insulin (1 μM), triiodothyronine (T3) (1 nM), dexamethasone (2 μg/ml) and IBMX (500 μM). After 3 days, the media was changed, and cells were maintained in DMEM 10% FBS supplemented with T3 (1 nM). T37i cells were induced to differentiate by treating confluent cells with insulin (20 μM) and T3 (2 nM) for the duration of differentiation. Triglycerides were extracted post-differentiation using the method of Folch [32]. Briefly, cells were scraped in 600 μl PBS and transferred to a glass tube. After addition of 3 ml of a chloroform:methanol (2:1) mixture, the tubes were vortexed and centrifuged for 10 min. The upper aqueous phase was discarded. The remaining lower phase was washed once with water, evaporated, dissolved in 100 μl isopropanol, and evaluated for triglycerides content using a standard assay kit (Thermo Scientific, TR22421). For Oil red O staining, cells were fixed with 4% paraformaldehyde (PFA) for 30 min at 37 °C, washed with PBS and stained for at least 30 min with Oil red O. Cells were then washed with PBS and imaged using an Olympus BX60 microscope (Tokyo, Japan).

### 2.4 Animal Care

All experimental protocols were approved by the Animal Ethics Committee of Université Laval (CPAUL) and following the guidelines of the Canadian Council on Animal Care. All mice were on a C57BL/6J background. *Deptor* floxed mice were generated from our group, as described previously [28]. *Myf5-*cre mice were purchased from The Jackson Laboratory (Stock number 007893). *Ucp1*-cre^ERT2^ were produced and kindly provided by the group of Christian Wolfrum [33]. To induce the deletion of DEPTOR in BAT of adult mice, *Deptor* floxed mice crossed with *Ucp1*-cre^ERT2^ were injected intraperitoneally with tamoxifen (0.150 mg/g body weight) three times over a period of 7 days. All mice were maintained on a 12:12-h light–dark cycle (lights on 0600–1800), while individually housed in ventilated cages at an ambient temperature (23 ± 1 °C), cold (10 ± 1 °C) or thermoneutrality (30 ± 1 °C). The animals were fed a normal chow diet. Rectal temperature was measured in mice using a digital thermometer with a precision of 0.1 °C (Mansfield, Montreal, QC, Canada). All mice were sacrificed, and tissues were collected at the same time of day.

### 2.5 Blood metabolite measurements

Glycemia was measured on fresh blood samples from the tail vein using a glucometer (Roche, Accu-Chek Performa). Plasma was prepared from blood samples collected using syringes conditioned with EDTA and stored at −80 °C. Plasma hormones and metabolites were measured according to the manufacturer's instructions using the following commercial kits: Insulin (Millipore Sigma, SRI-13K), Cholesterol (Randox laboratories, CH200), Triglycerides (Thermo Fisher Scientific, TR22421) and NEFAs (Wako, 999–34691, 995–34791, 991–34891, 993–35191, and 276–76491).

### 2.6 Tissue processing and cell size measurement

Brown adipose tissue samples were fixed during 48 h in 10% formalin at 4 °C. Tissues were next dehydrated, embedded in paraffin, and cut into 10 μm-thick sections. Sections were stained with hematoxylin and eosin (H&E). All pictures were taken on an Olympus BX60 microscope (Tokyo, Japan).

### 2.7 Quantitative real-time PCR

Total mRNA was isolated from tissues using the RNeasy Lipid Tissue Mini Kit (Qiagen, 74104). The RNA concentrations were estimated from absorbance at 260 nm. cDNA synthesis was performed using the iScript™ Advanced cDNA Synthesis Kit for RT-qPCR (Bio-Rad, Mississauga, ON, Canada). mRNA extraction and cDNA synthesis were performed following the manufacturer's instructions. cDNA was diluted in DNase-free water (1:15) before quantification by real-time PCR. mRNA transcript levels were measured in duplicate samples using a CFX96 or CFX384 touch™ real-time PCR (Bio-Rad, Mississauga, ON, Canada). Chemical detection of the PCR products was achieved with SYBR Green (Bio-Rad, Mississauga, ON, Canada). At the end of each run, melt curve analyses were performed, and representative samples of each experimental group were run on agarose gel to ensure the specificity of the amplification. Gene expression was corrected for the expression level of reference gene *Arbp*. The following primers were used.

**Table:** 

Gene	Forward primer	Reverse primer
*Arbp*	AGAAACTGCTGCCTCACATC	CATCACTCAGAATTTCAATGG
*Dio2*	CAGTGTGGTGCACGTCTCCAATC	TGAACCAAAGTTGACCACCAG
*Deptor*	AGCAGAGAGAGCTGGAACGC	CAGAGGCCTCCTTATGTTCA
*Fabp4*	GACGACAGGAAGGTGAAGAG	ACATTCCACCACCAGCTTGT
*Fasn*	CTGGCCCCGGAGTCGCTTGAGTATA	GGAGCCTCCGAAGCCAAATGA
*Fsp27*	GGGCAGAAGTGGAAGCCCCC	GAGGGCTTGGCCTTGGCAGG
*Pgc1a*	AAGATCAAGGTCCCCAGGCAGTAG	TGTCCGCGTTGTGTCAGGTC
*Pparg2*	ACTGCCTATGAGCACTTCAC	CAATCGGATGGTTCTTCGGA
*Pras40*	GACAGAAGCCCGATCGTCAG	ATTTCCGCTTCAGCTTCTGGA
*Prdm16*	CCAGATGTCAGCCATAGAAAC	CACAGTACTTGCACCTGTATGG
*Redd1*	CTGACGCTAAGTACCGGCTT	ACAGTCCTTCAGTCCTTGCC
*Scd1*	GCCCACCACAAGTTCTCAGA	GGGCGATATCCATAGAGATG
*Ucp1*	GCAGTGTTCATTGGGCAGCC	GGACATCGCACAGCTTGGTAC

### 2.8 Western blotting

Cells were rinsed twice with ice-cold PBS before lysis. Cells were lysed with Triton-X 100 containing lysis buffer (50 mM HEPES, pH 7.4, 2 mM EDTA, 10 mM sodium pyrophosphate, 10 mM sodium glycerophosphate, 40 mM NaCl, 50 mM NaF, 2 mM sodium orthovanadate, 1% Triton-X 100, and one tablet of EDTA-free protease inhibitors Roche per 25 ml). Tissues were homogenized with the same buffer supplemented with 0.1% sodium lauryl sulfate and 1% sodium deoxycholate. Cells and tissues were rotated at 4 °C for 10 min and then the soluble fractions of cell lysates were isolated by centrifugation at maximum speed for 10 min in a microcentrifuge. Protein levels were then quantified using Bradford reagents. Protein extracts were diluted in sample buffer, denaturated by heat (95 °C) for 10 min and loaded on precast gels (Life Technologies). Proteins were transferred to PVDF membranes blocked in 5% milk diluted in PBS-Tween and incubated with their primary antibody overnight at 4 °C. The following antibodies were used: ACTIN [Cell Signaling Technology, #4967, dilution 1:1000], Akt (pan) [Cell Signaling Technology, #4691, dilution 1:1000], Phospho-Akt (Ser473) [Cell Signaling Technology, #9271, dilution 1:1000], Phospho-Akt (Thr308) [Cell Signaling Technology, #2965, dilution 1:1000], DEPTOR [Novus Biologicals, #NBP1-49674, dilution 1:1000], S6 [Cell Signaling Technology, #2217, dilution 1:1000], Phospho- S6 (Ser240/244) [Cell Signaling Technology, #5364, dilution 1:1000]. Secondary antibodies were purchased from Cell signaling technology.

### 2.9 Statistics

All the statistical analyses were performed using GraphPad Prism 9. A detailed description of the statistical methods used for each experiments is presented in the figure legends.

## 3 Results

### 3.1 DEPTOR is highly expressed in BAT

**Figure 1:**
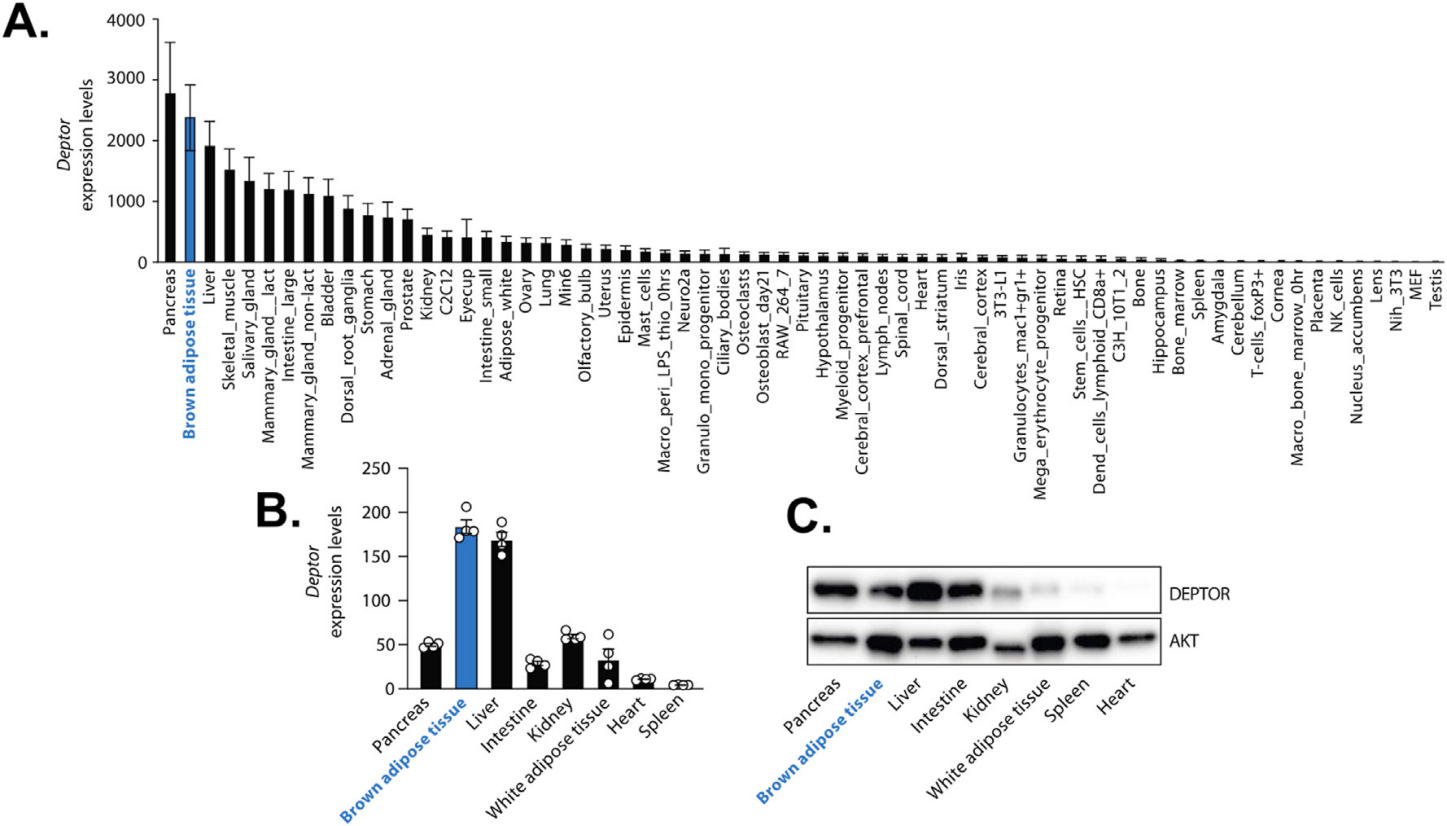
**DEPTOR is highly expressed in brown adipose tissue in mice. (A)** Gene expression profile comparing the expression of *Deptor* in various mouse tissues and cell types. The microarray data used in this panel are available through NCBI's Gene Expression Omnibus (accession number GSE10246). Data from five independent probe sets (n = 5) were used to calculate the average of *Deptor* expression in each tissue and cell type. **(B)** RNA was harvested from the indicated mouse tissues and *Deptor* expression was measured by RT-qPCR. This experiment was performed using tissues collected from 4 C57BL6/J wild-type male mice (n = 4). This experiment was performed in another cohort of mice and the same results were observed. **(C)** Proteins were extracted from mouse tissues and western blot analyses were performed. This experiment was reproduced twice and representative samples are presented. (For interpretation of the references to color in this figure legend, the reader is referred to the Web version of this article.)

To explore the physiological functions of DEPTOR, we have analyzed its distribution in mouse using BioGPS, an online resource that displays gene expression patterns from more than 90 tissues and cell types [34,35]. These microarrays data, available through NCBI's Gene Expression Omnibus (GEO accession number GSE10246), are commonly used to compare the expression profile of any given gene between a broad panel of tissues and cells. Using this tool, we found that *Deptor* expression is widely distributed, with some tissues/cell types showing nearly 350-fold higher *Deptor* levels compared to others (Figure 1A). Interestingly, this analysis revealed that *Deptor* expression is particularly high in several metabolic tissues, including BAT (Figure1A). Quantitative RT-PCR and western blot analyses confirmed these findings (Figure 1B,C), suggesting that DEPTOR might play a prominent role in regulating BAT function in mice.

### 3.2 DEPTOR expression is dynamically regulated in BAT in response to cold

**Figure 2:**
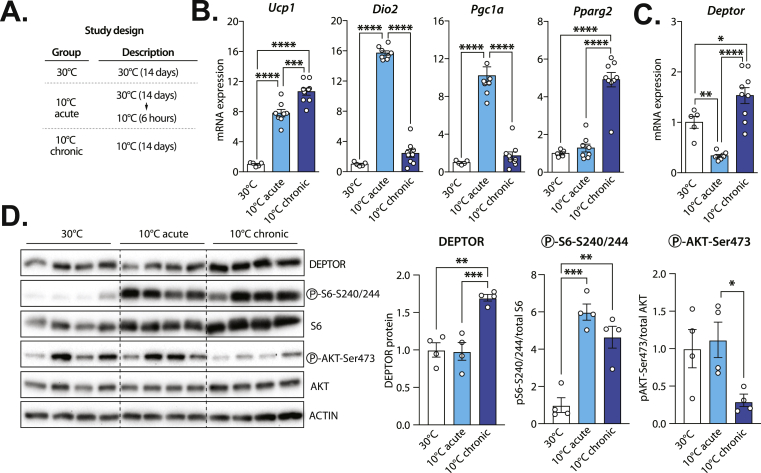
**DEPTOR expression is dynamically regulated by cold in brown adipose tissue. (A)** Overview of the experimental design. Briefly, a group of male mice was kept at thermoneutrality for 14 days (30 °C, n = 5). Another group of mice kept at thermoneutrality for 14 days was acutely exposed to cold for 6 h (10 °C acute, n = 8). Finally, one group of mice was exposed to cold for 14 days (10 °C chronic, n = 9). Because food intake elevates circulating levels of insulin and nutrients, all the mice were fasted for 6 h before sacrifice to avoid any contribution of these factors to the modulation of mTOR signaling. **(B–C)** RNA was extracted from BAT and gene expression was measured by RT-qPCR. **(D)** Proteins were extracted from BAT samples (n = 4) collected in the experiment described in A and western blots were performed for the indicated proteins. Quantification of the western blots is presented on the right side of the panel. In all panels, data represent the mean ± SEM. In panels B, C, and D, significance was determined using One-way ANOVA with Tukey's multiple-comparisons test (∗*p* < 0.05, ∗∗*p* < 0.01, ∗∗∗*p* < 0.001, ∗∗∗∗*p* < 0.0001). (For interpretation of the references to color in this figure legend, the reader is referred to the Web version of this article.)

Brown adipose tissue activates thermogenesis in response to cold challenge in rodents. We and others have shown that mTOR signalling is regulated by cold and that inactivating this pathway severely impairs BAT function in mice [[14], [15], [16], [17], [18],[20], [21], [22]]. Because DEPTOR serves as an important modulator of mTOR and considering its high levels in BAT (Figure1), we next analyzed its expression levels in response to acute (6h, 10 °C) and chronic (14 days, 10 °C) cold challenges (Figure 2A). Confirming the validity of our experimental approach, cold increased the expression of key thermogenic and lipogenic effectors in BAT (Figure2B). As shown in Figure 2C, *Deptor* expression was dynamically regulated in BAT upon cold challenges. In details, *Deptor* levels were decreased in response to acute cold but increased following chronic cold exposure (Figure2C). Western blot analyses next revealed that, despite the decrease in *Deptor* mRNA expression, acute cold did not impact DEPTOR protein levels (Figure2D), indicating that more time might be needed to control DEPTOR protein in BAT upon acute cold challenge. On the other hand, and in accordance with the mRNA expression data, we observed a significant rise in DEPTOR protein levels in BAT of mice exposed to cold for 14 days (Figure2D). Altogether, these results suggest that DEPTOR, a protein highly expressed in BAT, might play a role in regulating BAT recruitment and activation in response to cold challenge.

In the study described above, we found that the variations in DEPTOR levels were accompanied with important changes in the phosphorylation state of key effectors of the mTOR signaling pathway. In accordance with previous work, a striking rise in S6 phosphorylation, a downstream target of mTORC1, was measured in BAT following acute and chronic cold stimulation (Figure2D). Interestingly, the total level of S6 was also induced, which supports the importance of sustained protein synthesis for the adaptation of BAT during cold exposure. Chronic activation of mTORC1 activates several feedback loops that repress PI3K activity and Akt phosphorylation. In line with these findings, we observed that cold-mediated increase in mTORC1 was linked to a severe repression of Akt phosphorylation in BAT of mice chronically exposed to cold (Figure2D). These results indicate that mTOR signaling and DEPTOR levels are dynamically regulated in BAT in response to acute and chronic cold.

### 3.3 DEPTOR expression is induced during brown fat cell development

**Figure 3:**
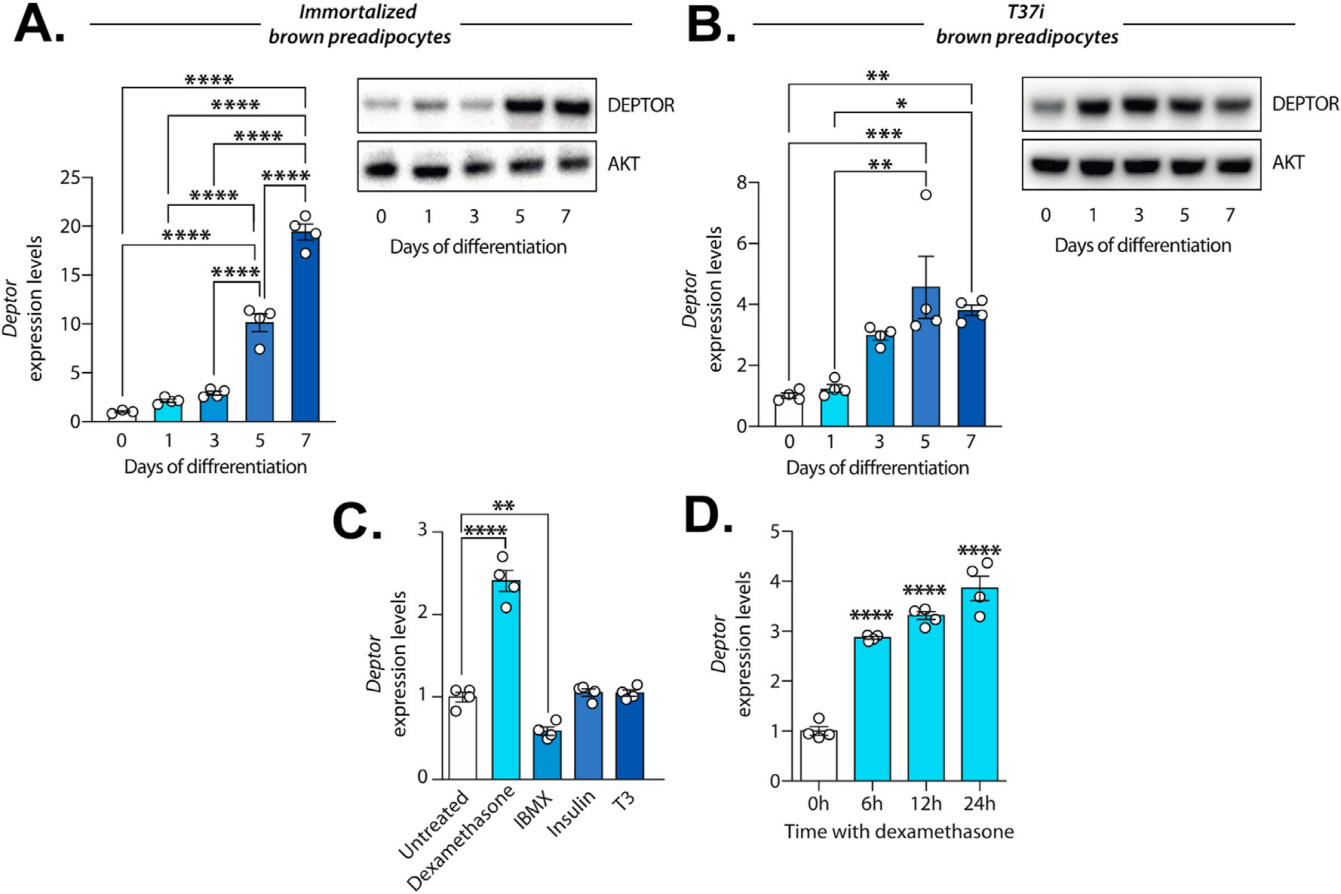
**DEPTOR expression is induced during brown fat cell development *in vitro*. (A)** Immortalized brown preadipocytes were induced to differentiate using an established adipogenic cocktail. RNA and proteins were harvested at the indicated times. *Deptor* mRNA expression and DEPTOR protein levels were measured by RT-qPCR (left side) and Western blotting (right side) respectively. Quantification of *Deptor* expression was performed on 4 independent samples per condition (n = 4). Representative blots from 2 independent experiments are presented on the right side of the panel. **(B)** T37i preadipocytes were induced to differentiate using an established adipogenic cocktail. RNA and proteins were harvested at the indicated times. *Deptor* mRNA expression and DEPTOR protein levels were measured by RT-qPCR (left side) and Western blotting (right side) respectively. Quantification of *Deptor* expression was performed on 4 independent samples per condition (n = 4). Representative blots from 3 independent experiments are presented on the right side of the panel. **(C)** Immortalized brown preadipocytes were treated with either dexamethasone (5 μM), IBMX (500 μM), insulin (5 μg/ml) or T3 (1 nM) for 12 h (n = 4/treatment). RNA was harvested and *Deptor* expression was measured by RT-qPCR. **(D)** Immortalized brown preadipocytes were treated with dexamethasone (5 μM) for 6, 12 and 24 h (n = 4/time). RNA was harvested and *Deptor* expression was measured by RT-qPCR. In all panels, data represent the mean ± SEM. Significance was determined using One-way ANOVA with Tukey's multiple-comparisons test (∗*p* < 0.05, ∗∗*p* < 0.01, ∗∗∗*p* < 0.001, ∗∗∗∗*p* < 0.0001). In panel C and D, significant differences versus the control condition are presented. (For interpretation of the references to color in this figure legend, the reader is referred to the Web version of this article.)

Chronic cold exposure triggers the expansion of BAT in mice and humans, a phenomenon linked to the recruitment of preadipocytes and their differentiation in mature fat cells [9,36]. As shown above, we observed that chronic cold was linked to an elevation in DEPTOR levels. To test whether DEPTOR is regulated upon brown fat cell development, immortalized brown preadipocytes were induced to differentiate *in vitro* using an established adipogenic cocktail. As shown in Figure 3A, we found that *Deptor* mRNA levels were gradually induced upon differentiation. This increase was approximately of 20-fold compared to preadipocytes prior to differentiation. An important rise in DEPTOR protein was also observed in terminally differentiated adipocytes (Figure 3A). Importantly, these observations were also reproduced using T37i cells, another cell line commonly used to study brown fat cell development *in vitro* (Figure 3B).

We next took advantage of the *in vitro* adipogenesis systems to investigate the mechanisms controlling *Deptor* expression during brown fat cell development. Immortalized preadipocytes were acutely treated with individual components of the adipogenic cocktail (i.e. dexamethasone, 3-isobutyl-1-methylxanthine (IBMX), insulin or T3) and the expression of *Deptor* was measured by qPCR. As presented in Figure 3C, dexamethasone, a synthetic glucocorticoid, is the only component of the cocktail that significantly increased *Deptor* expression. We found that IBMX slightly reduced *Deptor* levels whereas insulin and T3 had no effect. Time-response experiment next showed that dexamethasone increased *Deptor* expression within 6 h of treatment and that this effect was sustained over time (Figure3D). Altogether, these results indicate that DEPTOR is induced upon brown fat cell development and that glucocorticoid signaling plays a prominent role in this effect. These results also suggest that DEPTOR expression might participate to BAT development and expansion during cold stimulation.

### 3.4 DEPTOR depletion impairs brown fat cell development *in vitro*

**Figure 4:**
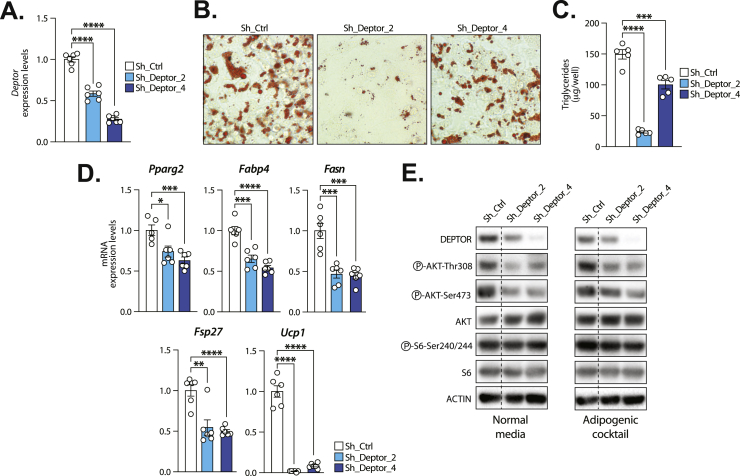
**DEPTOR depletion impairs brown fat cell development *in vitro*. (A)** T37i cells were transduced with lentivirus expressing control shRNA (shCtrl) or shRNA to knockdown DEPTOR (shDeptor_2 or shDeptor_4). Following puromycin selection, RNA was extracted and *Deptor* mRNA expression levels were measured by RT-qPCR. Quantification of *Deptor* expression was performed on 6 independent samples per condition (n = 6). **(B)** T37i cells expressing shCtrl, shDeptor_2 or shDeptor_4 were induced to differentiate using an established adipogenic cocktail. Oil-red O staining was performed 5 days after the induction of adipogenesis. Representative pictures are shown. **(C)** Triglycerides were extracted and quantified from the experiment described in B. This experiment was performed on 5 independent samples per condition (n = 5). **(D)** RNA was extracted from the experiment described in C and the expression of adipogenic and thermogenic genes was measured by RT-qPCR. This experiment was performed on 5-6 independent samples per condition (n = 5-6). **(E)** T37i cells expressing shCtrl, shDeptor_2 or shDeptor_4 were washed with PBS and then exposed to either normal media or the adipogenic cocktail for 6 h. Proteins were extracted and western blot analyses were performed for the indicated proteins. This experiment was reproduced three times. The quantification of phosphoproteins is presented in Figure S1C. In all panels, data represent the mean ± SEM. Significance was determined using two-tailed unpaired *t* test vs shCtrl (∗*p* < 0.05, ∗∗*p* < 0.01, ∗∗∗*p* < 0.001, ∗∗∗∗*p* < 0.0001). (For interpretation of the references to color in this figure legend, the reader is referred to the Web version of this article.)

In order to define the functional role of DEPTOR in brown fat cell development, brown preadipocytes were transduced with lentivirus expressing short-hairpin RNA (shRNA) to repress DEPTOR expression. As presented in Figure 4A and S1A, we identified independent shRNA that efficiently decreased *Deptor* levels. These cells were next induced to differentiate using an established adipogenic cocktail. We found that DEPTOR depletion severely impaired fat cell development *in vitro* (Figure 4B and S1B). We observed a clear reduction in lipid droplet formation in response to DEPTOR knockdown. A corresponding decrease in triglyceride accumulation was also measured in DEPTOR depleted cells (Figure 4C). Consistent with the decrease in lipid accumulation in brown preadipocytes, we found a significant reduction in the expression of several markers of terminal differentiation in cells depleted from DEPTOR, namely *peroxisome proliferator-activated receptor gamma 2* (*Pparg2*), *Fatty acid binding protein 4* (*Fabp4*), *Fatty acid synthase* (*Fasn*), and *Fat-specific protein 27* (*Fsp27*) (Figure 4D). A severe depletion in the expression of *Ucp1* was also measured in response to DEPTOR knockdown. These results support a key role for DEPTOR in promoting brown fat cell development *in vitro.*

Previous studies showed that DEPTOR positively controls white fat cell development by dampening mTORC1-mediated feedback inhibition of insulin signaling and by promoting Akt activation [29]. To define whether DEPTOR controls brown adipogenesis through a similar mechanism, the phosphorylation state of central components of the mTOR signaling pathway was measured in DEPTOR-depleted brown preadipocytes exposed or not to the adipogenic cocktail. Consistent with previous work, we found that DEPTOR depletion severely repressed the phosphorylation of Akt on both Ser473 and Thr308 (Figure 4E and Fig.S1C). DEPTOR knockdown did not impact the phosphorylation of S6 in preadipocytes. Overall, these findings indicate that the adipogenic defect observed in response to DEPTOR depletion is associated with the rewiring of mTOR signaling in brown preadipocytes.

### 3.5 Loss of DEPTOR in *Myf5-*expressing cells minimally impacts BAT development, recruitment, and thermogenic activation

**Figure 5:**
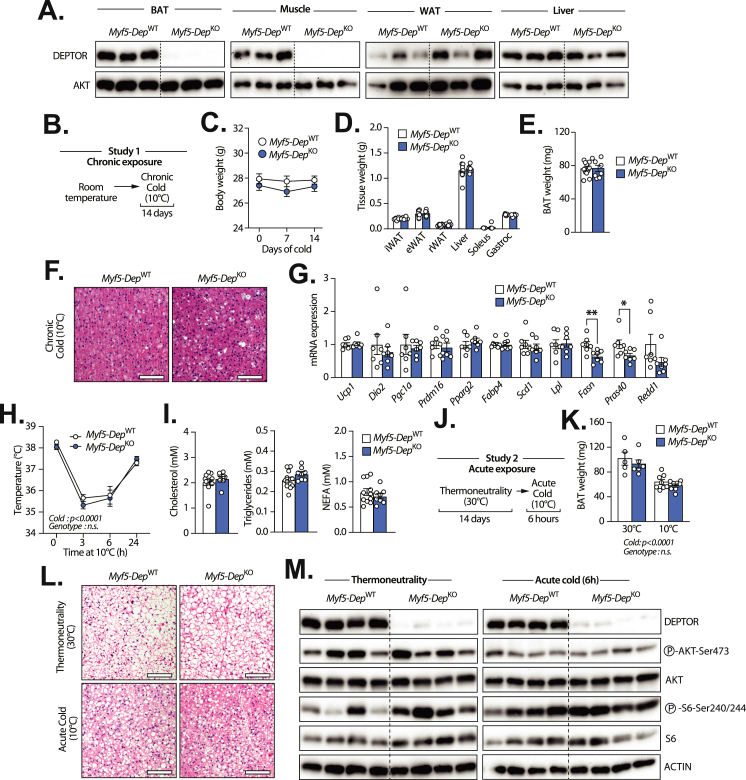
**Loss of DEPTOR in Myf5-expressing cells has limited impacts on BAT development, recruitment, and thermogenic activation. (A)** Proteins were extracted from tissues of *Myf5*-*Dep*^WT^ (n = 3) and *Myf5*-*Dep*^KO^ (n = 3) mice and western blot analyses were performed for the indicated proteins. The muscle presented is the gastrocnemius and the WAT is the inguinal adipose tissue. **(B)** Schematic presentation of the experimental approach aiming at defining the impact of chronic cold exposure in *Myf5*-*Dep*^WT^ and *Myf5*-*Dep*^KO^ mice. Male mice (12 week-old) were exposed to cold (10 °C) for 14 days and were then sacrificed. **(C)** Body weight and **(D)** tissue weight of *Myf5*-*Dep*^WT^ (n = 12) and *Myf5*-*Dep*^KO^ (n = 8) male mice exposed to cold for 14 days. In panel D, the following abbreviations are used: inguinal WAT (iWAT), epididymal WAT (eWAT), retroperitoneal WAT (rWAT). **(E)** Weight and **(F)** H&E-stained sections of BAT isolated from *Myf5*-*Dep*^WT^ (n = 12) and *Myf5*-*Dep*^KO^ (n = 8) male mice exposed to cold for 14 days. Representative pictures are presented in panel F (scale: 100 μM) **(G)** RNA was extracted from BAT of *Myf5*-*Dep*^WT^ (n = 7) and *Myf5*-*Dep*^KO^ (n = 7) male mice following cold exposure and the expression of adipogenic, lipogenic and thermogenic genes was measured by RT-qPCR. **(H)** Rectal temperature of *Myf5*-*Dep*^WT^ (n = 7) and *Myf5*-*Dep*^KO^ (n = 10) male mice acutely exposed to cold. **(I)** Circulating cholesterol, triglycerides, and non-esterified fatty acids (NEFA) in *Myf5*-*Dep*^WT^ (n = 12) and *Myf5*-*Dep*^KO^ (n = 8) following chronic cold exposure. **(J)** Schematic presentation of the experimental approach aiming at defining the impact of thermoneutrality and acute cold exposure in *Myf5*-*Dep*^WT^ and *Myf5*-*Dep*^KO^ mice. Male mice (12 week-old) were housed at thermoneutrality (30 °C) for 14 days and were sacrificed or acutely exposed to cold (10 °C) for 6 h. **(K)** Weight and **(L)** H&E-stained sections of BAT isolated from *Myf5*-*Dep*^WT^ (n = 5 [30 °C]; n = 8 [10 °C]) and *Myf5*-*Dep*^KO^ (n = 6 [30 °C]; n = 8 [10 °C]) male mice treated as described in J. Representative pictures are presented in panel L (scale: 100 μM). **(M)** Proteins were extracted from tissues of *Myf5*-*Dep*^WT^ and *Myf5*-*Dep*^KO^ male mice housed at thermoneutrality (30 °C) for 2 weeks (*Myf5*-*Dep*^WT^ [n = 5] and *Myf5*-*Dep*^KO^ [n = 5]) or after acute cold exposure (10 °C, 6 h) (*Myf5*-*Dep*^WT^ [n = 7] and *Myf5*-*Dep*^KO^ [n = 7]) and western blot analyses were performed for the indicated proteins. All the mice were fasted for 6 h before sacrifice. Representative samples are shown. The quantification of the western blots is presented in Figure S2. In all panels, data represent the mean ± SEM. In panel C, D, E, G, and I, significance was determined using two-tailed unpaired *t* test (∗*p* < 0.05, ∗∗*p* < 0.01, ∗∗∗*p* < 0.001, ∗∗∗∗*p* < 0.0001). In panel H and K, significance was determined using Two-way ANOVA with Sidak's multiple-comparisons test (∗*p* < 0.05, ∗∗*p* < 0.01, ∗∗∗*p* < 0.001, ∗∗∗∗*p* < 0.0001).

DEPTOR expression is elevated in BAT and its levels are induced by chronic cold exposure. *In vitro*, we showed that DEPTOR knockdown severely impairs the adipogenic conversion of brown preadipocytes. To test the physiological impact of DEPTOR loss on BAT development, recruitment, and thermogenic activation *in vivo*, DEPTOR floxed mice [28] were crossed with *Myf5*-Cre mice, a model allowing the conditional recombination of LoxP site in the mesenchymal precursor cells giving rise to BAT and skeletal muscle [21,37,38]. From now, we refer to wild-type mice as *Myf5*-*Dep*^WT^ and knockout mice as *Myf5*-*Dep*^KO^. As expected, we found that the crossing strategy led to the conditional deletion of DEPTOR in BAT (Figure 5A). DEPTOR was also deleted in muscles, but not in other tissues including white adipose tissue (WAT) and the liver (Figure 5A). We noticed no clear physiological difference between *Myf5*-*Dep*^WT^ and *Myf5*-*Dep*^KO^ mice. In a first set of experiments, mice were housed at room temperature and then chronically exposed to cold for 14 days (Figure 5B). Body weight and tissue weight did not differ between the genotypes (Figure 5C,D). Unexpectedly, we found no impact of DEPTOR loss on BAT size (Figure 5E). Histological analyses of BAT revealed no difference between *Myf5*-*Dep*^WT^ and *Myf5*-*Dep*^KO^ (Figure 5F). The expression of lipogenic and thermogenic genes was minimally affected by DEPTOR loss (Figure 5G). The only gene that was significantly reduced in BAT of *Myf5*-*Dep*^KO^ mice is *Fas*. To functionally test the impact of DEPTOR loss on thermogenesis, we next measured body temperature in *Myf5*-*Dep*^WT^ and *Myf5*-*Dep*^KO^ upon cold exposure. As depicted in Figure 5H, DEPTOR loss did not affect rectal temperature in response to cold. Consistent with the mild phenotype of *Myf5*-*Dep*^KO^ mice, we measured no changes in circulating cholesterol, triglycerides, and non-esterified fatty acids (NEFAs) compared to wild-type mice (Figure 5I). We next tested in complementary studies whether DEPTOR loss could acutely impact BAT in mice transitioning from a thermoneutral (30 °C) to a cold environment (10 °C) (Figure 5J). As previously reported [15], we found that BAT mass was higher in mice housed at thermoneutrality compared to mice acutely exposed to cold (Figure 5K), an effect directly linked to higher lipid content in BAT of mice exposed to a thermoneutral environment (Figure 5L). Consistent with the results presented above, neither BAT weight nor histological appearance differed between *Myf5*-*Dep*^WT^ and *Myf5*-*Dep*^KO^ (Figure 5K,L). As presented in Figure 4, DEPTOR knockdown severely impairs adipogenesis *in vitro*, an effect associated with the rewiring of mTOR signaling and the inhibition of Akt. Contrary to what we observed in response to DEPTOR depletion in preadipocytes *in vitro*, we found no difference in the phosphorylation of Akt between *Myf5*-*Dep*^WT^ and *Myf5*-*Dep*^KO^ (Figure 5M and Figure S2). Altogether, these results indicate that DEPTOR loss in *Myf5* expressing cells did not affect mTOR signalling in BAT and had minimal impacts on brown adipocyte recruitment, development, and activation in mice.

### 3.6 DEPTOR loss in mature brown fat cells minimally affects BAT structure and function

**Figure 6:**
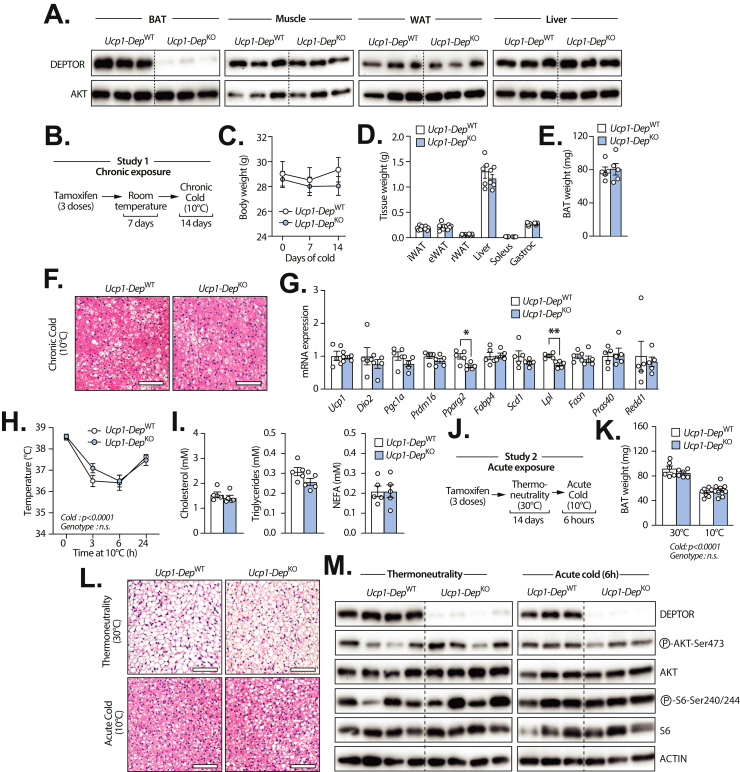
**Loss of DEPTOR in mature brown fat cells has limited impacts on brown adipose tissue structure and function.** Proteins were extracted from tissues of *Ucp1*-*Dep*^WT^ (n = 3) and *Ucp1*-*Dep*^KO^ (n = 3) mice and western blot analyses were performed for the indicated proteins. The muscle presented is the gastrocnemius and the WAT is the inguinal adipose tissue. **(B)** Schematic presentation of the experimental approach aiming at defining the impact of chronic cold exposure in *Ucp1*-*Dep*^WT^ and *Ucp1*-*Dep*^KO^ mice. Male mice (13 week-old) were exposed to cold (10 °C) for 14 days and were then sacrificed. **(C)** Body weight and **(D)** tissue weight of *Ucp1*-*Dep*^WT^ (n = 5) and *Ucp1*-*Dep*^KO^ (n = 5) male mice exposed to cold for 14 days. In panel D, the following abbreviations are used: inguinal WAT (iWAT), epididymal WAT (eWAT), retroperitoneal WAT (rWAT). **(E)** Weight and **(F)** H&E-stained sections of BAT isolated from *Ucp1*-*Dep*^WT^ (n = 5) and *Ucp1*-*Dep*^KO^ (n = 5) male mice exposed to cold for 14 days. Representative pictures are presented in panel F (scale: 100 μM). **(G)** RNA was extracted from BAT of *Ucp1*-*Dep*^WT^ (n = 5) and *Ucp1*-*Dep*^KO^ (n = 5) male mice following cold exposure and the expression of adipogenic, lipogenic and thermogenic genes was measured by RT-qPCR. **(H)** Rectal temperature of *Ucp1*-*Dep*^WT^ (n = 5) and *Ucp1*-*Dep*^KO^ (n = 5) male mice acutely exposed to cold. **(I)** Circulating cholesterol, triglycerides, and non-esterified fatty acids (NEFA) in *Ucp1*-*Dep*^WT^ (n = 5) and *Ucp1*-*Dep*^KO^ (n = 5) following chronic cold exposure. **(J)** Schematic presentation of the experimental approach aiming at defining the impact of thermoneutrality and acute cold exposure in *Ucp1*-*Dep*^WT^ and *Ucp1*-*Dep*^KO^ mice. Male mice (13-week-old) were housed at thermoneutrality (30 °C) for 14 days and were sacrificed or acutely exposed to cold (10 °C) for 6 h. **(K)** Weight and **(L)** H&E-stained sections of BAT isolated from *Ucp1*-*Dep*^WT^ (n = 6 [30 °C]; n = 7 [10 °C]) and *Ucp1*-*Dep*^KO^ (n = 6 [30 °C]; n = 10 [10 °C]) male mice treated as described in J. Representative pictures are presented in panel L (scale: 100 μM). **(M)**. Proteins were extracted from tissues of *Ucp1*-*Dep*^WT^ and *Ucp1*-*Dep*^KO^ male mice housed at thermoneutrality (30 °C) for 2 weeks (*Ucp1*-*Dep*^WT^ [n = 6] and *Ucp1*-*Dep*^KO^ [n = 6]) or after acute cold exposure (10 °C, 6 h) (*Ucp1*-*Dep*^WT^ [n = 6] and *Ucp1*-*Dep*^KO^ [n = 6]) and western blot analyses were performed for the indicated proteins. All the mice were fasted for 6 h before sacrifice. Representative samples are shown. The quantification of the western blots is presented in Figure S3. In all panels, data represent the mean ± SEM. In panel C, D, E, G, and I, significance was determined using two-tailed unpaired *t* test (∗*p* < 0.05, ∗∗*p* < 0.01, ∗∗∗*p* < 0.001, ∗∗∗∗*p* < 0.0001). In panel H and K, significance was determined using Two-way ANOVA with Sidak's multiple-comparisons test (∗*p* < 0.05, ∗∗*p* < 0.01, ∗∗∗*p* < 0.001, ∗∗∗∗*p* < 0.0001). (For interpretation of the references to color in this figure legend, the reader is referred to the Web version of this article.)

The absence of phenotype in *Myf5*-*Dep*^KO^ was unexpected considering the deep impact of DEPTOR depletion on brown preadipocyte differentiation *in vitro*. We reasoned that this may have been the result of compensation linked to the chronic loss of DEPTOR in *Myf5*-expressing cells. To circumvent this problem, DEPTOR floxed mice were next bred to *Ucp1-*Cre^ERT2^ mice, a model that specifically allows recombination of floxed alleles in mature brown adipocytes upon tamoxifen administration [33,39]. As expected, administration of tamoxifen to these mice led to the conditional deletion of DEPTOR specifically in BAT, and not in any other tissues (Figure 6A). From now, we refer to wild-type mice as *Ucp1*-*Dep*^WT^ and knockout mice as *Ucp1*-*Dep*^KO^. In a first experiment, mice were injected with tamoxifen, housed at room temperature for 7 days and then chronically exposed to cold for 14 days (Figure 6B). As observed above, DEPTOR loss did not have any impact on body weight and tissue weight (Figure 6C,D). The weight and the histology of BAT was not different between *Ucp1*-*Dep*^WT^ and *Ucp1*-*Dep*^KO^ mice (Figure 6E,F). The expression of thermogenic genes was not affected by DEPTOR loss (Figure 6G). However, we found a slight but significant reduction in the expression of *Pparg2* and *Lipoprotein lipase* (*Lpl)* in BAT of *Ucp1*-*Dep*^KO^ mice (Figure 6G). Despite these subtle changes, DEPTOR loss did not affect body temperature in mice acutely exposed to cold (Figure 6H). We also found no difference in circulating lipids between *Ucp1*-*Dep*^WT^ and *Ucp1*-*Dep*^KO^ mice (Figure 6I). As described above, we next tested the impact of DEPTOR loss in mice transitioning from a thermoneutral (30 °C) to a cold environment (10 °C) (Figure 6J). We observed that BAT weight was reduced in response to acute cold exposure, an effect that was similar between *Ucp1*-*Dep*^WT^ and *Ucp1*-*Dep*^KO^ mice (Figure 6K). Histology analyses of BAT did not reveal any difference between the genotypes (Figure 6L). As observed in *Myf5*-*Dep*^KO^ mice, we found no effect of DEPTOR deletion on the phosphorylation of key effectors of the mTOR signaling pathway (Figure 6M and Figure S3). Altogether, these observations show that DEPTOR loss in mature brown adipocyte minimally impacts BAT structure and thermogenesis in mice. These findings support the observations made in *Myf5*-*Dep*^KO^ mice and indicate that DEPTOR is dispensable for BAT development and function in mice.

## 4 Discussion

Over the last years, the mTOR signaling pathway was identified as a key node modulating BAT development, recruitment, and thermogenic activation in mice. We and others have shown that mTORC1 activity is induced by acute and chronic cold exposure and β3-adrenergic receptor stimulation [[14], [15], [16], [17], [18]]. Supporting the importance of mTORC1 in BAT, adipose-specific deletion of RAPTOR, a key component of mTORC1, reduces BAT size and prevents cold-induced BAT expansion and metabolic adaptation in mice [15,16,19]. mTORC2 activity is also acutely activated by cold in brown adipocytes [14,15,17,18,20]. Loss of function studies showed that mTORC2 controls various metabolic pathways impacting lipogenesis and adipogenesis in BAT [14,17,[20], [21], [22]]. DEPTOR is a conserved protein that interacts with mTOR complexes to regulate their function [23]. Studies performed in various experimental models show that DEPTOR often represses mTORC1 action to prevent the feedback inhibition of insulin signaling [24]. Whether DEPTOR impacts BAT function by modulating mTOR signaling was never tested. Here, we found that DEPTOR is highly expressed in BAT and that its levels are induced upon brown adipocyte differentiation and in response to chronic cold exposure. We show that DEPTOR depletion impairs adipogenesis *in vitro*, an effect associated with the inhibition of Akt and a reduction in the expression of key adipogenic markers. Conditional deletion of DEPTOR in BAT slightly reduced the expression of adipogenic and lipogenic genes but did not impact BAT recruitment and thermogenesis in mice. Altogether, our findings indicate that DEPTOR plays roles in brown fat cell development *in vitro* but that the impacts of its deletion in mice are limited.

DEPTOR expression is widely distributed in mice. Here, we report that DEPTOR levels are elevated in BAT, but also in several other insulin- and nutrient-sensitive tissues including the pancreas, liver, and intestine. These observations are particularly interesting owing to the importance of mTOR signaling in regulating key metabolic processes in these tissues [[40], [41], [42], [43], [44]]. In addition of being highly expressed in BAT, DEPTOR levels are also dynamically regulated by cold in this tissue. We found that *Deptor* transcription is rapidly decreased in BAT following cold stimulation, an effect that occurred concomitantly with the activation of mTORC1. This observation, that aligns with previous findings showing that *Deptor* expression inversely correlates with mTORC1 activity [23], suggests that mTORC1 auto amplifies its activity by decreasing the expression of its endogenous inhibitor. Interestingly, a significant decrease in *DEPTOR* expression was also observed in human thermogenic adipocytes acutely treated with norepinephrine *in vivo* (Figure S4) [45], indicating that this response is conserved in mice and humans. We propose that reducing DEPTOR levels might take place to maximize mTORC1 action upon acute cold challenge, an effect that is important to promote mitochondrial biogenesis, nucleotide synthesis, and oxidative metabolism in brown adipocytes [15].

The regulation of DEPTOR differed upon chronic cold exposure. We found that chronic cold led to a significant rise in DEPTOR levels in BAT. This effect occurred despite elevated mTORC1 activity, indicating that other regulatory processes are taking over to drive DEPTOR expression in this context. We observed that DEPTOR expression was induced during brown adipogenesis and that dexamethasone, a synthetic glucocorticoid, played a role in this effect. Supporting these findings, a glucocorticoid response element was previously identified upstream of *Deptor* gene and pharmacological inhibition of the glucocorticoid receptor was shown to repress *Deptor* expression [29]. Why is DEPTOR expression induced in response to chronic cold is a very interesting question. Numerous studies showed that DEPTOR overexpression represses mTORC1-mediated feedback inhibition of PI3K-Akt signaling [23,27,29,46,47]. Thus, the rise in DEPTOR levels might serve to counteract hyperactive mTORC1 to preserve basal Akt functions in response to chronic SNS stimulation. Because Akt controls central processes in BAT such as lipogenesis, adipogenesis and thermogenesis [14,22,48], preserving its activity is probably of great importance in supporting the long-term adaptation to cold temperatures.

We have previously reported a link between DEPTOR and white adipocyte development in mice [29]. In this study, we found that *Deptor* is part of a quantitative trait locus linked to obesity/leanness in mice, with DEPTOR levels being elevated in WAT of obese animals. Supporting a positive relationship between DEPTOR and white adipocyte development, mice overexpressing DEPTOR gained more weight in response to high-fat feeding. We also found that mouse embryonic fibroblast (MEFs) isolated from DEPTOR-inducible mice expressed higher adipogenic/lipogenic genes and accumulated more lipids when induced to differentiate [29]. As reported here in brown preadipocytes, DEPTOR knockdown impaired adipogenesis in 3T3-L1 cells. Importantly, this phenotype was also linked to the rewiring of mTOR signaling and the impairment in Akt activation [29]. Taken together, these results indicate that DEPTOR is a key protein regulating Akt activation during adipogenesis, and that this relation is conserved during the development of both white and brown preadipocytes.

Because DEPTOR depletion in brown preadipocytes severely repressed adipogenesis *in vitro*, we expected that its deletion would impair BAT development and thermogenesis in mice. However, contrary to our hypothesis, DEPTOR loss did not reduce BAT mass and did not alter the acute and chronic responses to cold *in vivo*. Importantly, these observations were made in two independent mouse models allowing the deletion of DEPTOR in brown adipose progenitors (*Myf5-Cre*) and mature brown fat cells (*Ucp1-Cre*^*ERT2*^). Why DEPTOR depletion severely repressed brown adipogenesis *in vitro* but remained without clear effects in mice is an intriguing question. Here, we show that DEPTOR knockdown in preadipocytes severely repressed Akt activity. An important reduction in the expression of adipogenic/lipogenic genes was also measured in this *in vitro* context. In DEPTOR null mice, we did not measure severe changes in signaling, but found a small decrease in the expression of few adipogenic/lipogenic genes in BAT. Clearly, these changes were not sufficient to impair BAT development and activation. Nevertheless, this partial overlap between *in vitro* and *in vivo* studies suggest that compensatory mechanisms might have been triggered in DEPTOR null mice to minimize the impact of DEPTOR loss. Supporting this possibility, we have previously reported that compensation occurs in the face of chronic DEPTOR deletion in mice [28]. For instance, even though DEPTOR overexpression was reported to promote WAT expansion in mice [29], whole-body DEPTOR null mice did not show defect in WAT development [28]. Also, in the current study, we found no effect of DEPTOR loss on the development of retroperitoneal WAT (rWAT), a tissue in which *Myf5*-Cre is expressed and efficiently recombines floxed alleles [49]. It is important to point out that, in addition to DEPTOR, other proteins were reported to repress mTORC1 function *in vivo* and *in vitro*. For instance, proline rich Akt substrate of 40 kDa (PRAS40) directly interacts with mTORC1 to repress its kinase activity [[50], [51], [52]]. Regulated in development and DNA damage response 1 (REDD1) is another protein that represses mTORC1 function in response to various stresses [[53], [54], [55]]. Here, we found that DEPTOR loss did not lead to a compensatory rise in the expression of either *Pras40* and *Redd1* (Figure 5, Figure 6G). However, we cannot exclude the possibility that the expression of other proteins fine-tuning mTOR signaling might have been altered to compensate the loss of DEPTOR in mice. Clearly, additional work is needed to define the precise mechanisms by which tissues adapt to the deletion of DEPTOR.

## 5 Conclusion

DEPTOR is highly expressed in BAT and its levels are dynamically regulated during brown fat cell development and upon cold exposure. Although DEPTOR depletion severely represses brown fat adipogenesis *in vitro*, its deletion is dispensable for BAT development, recruitment, and thermogenic activation in mice.

## Data Availability

No data was used for the research described in the article.
