# Supplementary material for: DEPTOR loss impairs brown adipocyte development in vitro but has limited impacts in mice
Source: Mol Metab. 2022 Dec 16;67:101660. doi: 10.1016/j.molmet.2022.101660 (PMC9827061; doi:10.1016/j.molmet.2022.101660)
Supplement: Multimedia component 1 [file mmc1.docx]

**­­**

**Figure S1. DEPTOR depletion impairs brown fat cell development *in vitro*. (A)** T37i cells were transduced with lentivirus expressing control shRNA (shCtrl) or shRNA to knockdown DEPTOR (shDeptor_6). Following puromycin selection, RNA was extracted and *Deptor* mRNA expression levels were measured by RT-qPCR. Quantification of *Deptor* expression was performed on 4 independent samples per condition (n=4). **(B)** T37i cells expressing shCtrl, shDeptor_6 were induced to differentiate using an established adipogenic cocktail. Oil-red O staining was performed 5 days after the induction of adipogenesis. **(C)** T37i cells expressing shCtrl, shDeptor_2 or shDeptor_4 were washed with PBS and then exposed to either normal media or the adipogenic cocktail for 6 hours. Proteins were extracted and western blot analyses were performed for the indicated proteins. This experiment was reproduced three times. The quantification of phosphoproteins is presented (n=3). In all panels, data represent the mean ± SEM. Significance was determined using two-tailed unpaired *t* test vs shCtrl (**p*<0.05, ***p*<0.01, ****p*<0.001, *****p*<0.0001). Non-significant effects (n.s.) are indicated on the graph.

**Figure S2. Loss of DEPTOR in Myf5-expressing cells has limited impacts on BAT development, recruitment, and thermogenic activation.** Quantification of the western blots presented in Figure 5M. Data represent the mean ± SEM. Significance was determined using two-tailed unpaired *t* test (**p*<0.05, ***p*<0.01, ****p*<0.001, *****p*<0.0001). Non-significant effects (n.s.) are indicated on the graph.

**Figure S3. Loss of DEPTOR in mature brown fat cells has limited impacts on brown adipose tissue structure and function.** Quantification of the western blots presented in Figure 6M. Data represent the mean ± SEM. Significance was determined using two-tailed unpaired *t* test (**p*<0.05, ***p*<0.01, ****p*<0.001, *****p*<0.0001). Non-significant effects (n.s.) are indicated on the graph.

**Figure S4. Norepinephrine treatment reduces *DEPTOR* expression in human thermogenic adipocytes.** The results presented in this figure were extracted from the supplementary information of a study previously published [45]. The GEO accession number for the RNA-seq data is GSE150119. Briefly, biopsies of supraclavicular adipose tissue were obtained from non-diabetic participants. Stromovascular cells were extracted by collagenase digestion and cells were differentiated and stimulated for 4h with norepinephrine (NE) before RNA sequencing (RNA-seq) (saline n=5, NE = 5). Statistical significance was calculated using paired, two-tailed *t* test. (**p*<0.05, ***p*<0.01, ****p*<0.001, *****p*<0.0001).
